# A highly stable prefusion RSV F vaccine derived from structural analysis of the fusion mechanism

**DOI:** 10.1038/ncomms9143

**Published:** 2015-09-03

**Authors:** Anders Krarup, Daphné Truan, Polina Furmanova-Hollenstein, Lies Bogaert, Pascale Bouchier, Ilona J. M. Bisschop, Myra N. Widjojoatmodjo, Roland Zahn, Hanneke Schuitemaker, Jason S. McLellan, Johannes P. M. Langedijk

**Affiliations:** 1 Janssen Infectious Diseases and Vaccines, Archimedesweg 4-6, Leiden 2333 CN, The Netherlands; 2 Department of Biochemistry, Geisel School of Medicine at Dartmouth, Hanover, New Hampshire 03755-3844, USA

## Abstract

Respiratory syncytial virus (RSV) causes acute lower respiratory tract infections and is the leading cause of infant hospitalizations. Recently, a promising vaccine antigen based on the RSV fusion protein (RSV F) stabilized in the native prefusion conformation has been described. Here we report alternative strategies to arrest RSV F in the prefusion conformation based on the prevention of hinge movements in the first refolding region and the elimination of proteolytic exposure of the fusion peptide. A limited number of unique mutations are identified that stabilize the prefusion conformation of RSV F and dramatically increase expression levels. This highly stable prefusion RSV F elicits neutralizing antibodies in cotton rats and induces complete protection against viral challenge. Moreover, the structural and biochemical analysis of the prefusion variants suggests a function for p27, the excised segment that precedes the fusion peptide in the polypeptide chain.

## Abstract (web summary)

Respiratory syncytial virus (RSV) is a highly contagious childhood pathogen of the respiratory tract for which no vaccine is currently available. Here the authors present a strategy to stabilize the RSV F protein in a prefusion conformation that can elicit a strong protective immune response in animal models.

## Graphical abstract

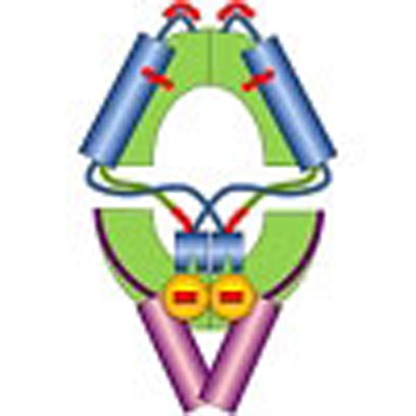

Respiratory syncytial virus (RSV) is a highly contagious childhood pathogen of the respiratory tract^1^. In children younger than 2 years, RSV accounts for ∼50% of the hospitalizations due to respiratory infections, with a peak of hospitalization occurring at 2–4 months of age^2^. It has been reported that almost all children have experienced infection with RSV by the age of two^3^, and repeated infection during life is attributed to low natural immunity. In the elderly, the RSV disease burden is similar to those caused by non-pandemic influenza A infections^4^.

A vaccine against RSV is currently not available, but is desired due to the high disease burden. The RSV fusion glycoprotein (RSV F) is an attractive vaccine antigen, since it is the principal target of RSV neutralizing antibodies in human sera^5^,^6^,^7^. A neutralizing monoclonal antibody against RSV F (palivizumab) can prevent severe disease and has been approved for prophylaxis in preterm infants^8^. RSV F fuses the viral and host cell membranes by irreversible protein refolding from the labile prefusion conformation to the stable postfusion conformation^9^. Structures of both conformations have been determined for RSV F^10^,^11^,^12^,^13^, as well as for the fusion proteins from related paramyxoviruses, providing insight into the mechanism of this complex fusion machine^14^,^15^,^16^. Like other class I fusion proteins, the inactive precursor, RSV F_0_, requires cleavage during intracellular maturation by a furin-like protease. RSV F contains two furin sites, which leads to three polypeptides: F2, p27 and F1, with the latter containing a hydrophobic fusion peptide at its N terminus (Fig. 1)^17^,^18^. To refold from the prefusion to the postfusion conformation, the refolding region 1 (RR1), which spans residues 137 through 216 and includes the fusion peptide and heptad repeat A (HRA), has to transform from an assembly of helices, loops and strands to a long continuous helix (Fig. 2). The fusion peptide, located at the N-terminal segment of RR1, is then able to extend away from the viral membrane and insert into the membrane of the target cell. Next, the refolding region 2 (RR2), which forms the C-terminal stem in the prefusion F spike and includes the heptad repeat B (HRB), relocates to the other side of the RSV F head. This allows HRB to bind the HRA coiled-coil trimer and form the six-helix bundle^17^,^18^. The formation of the RR1 coiled-coil and relocation of RR2 to complete the six-helix bundle are the most dramatic structural changes that occur during the refolding process.

Most *in vitro* RSV neutralizing antibodies in human sera are directed against the prefusion conformation^5^, but due to its instability the prefusion conformation has a propensity to prematurely refold into the stable postfusion conformation, both in solution and on the surface of the virions^10^,^19^. The stabilization of the RSV F protein has recently been achieved by the introduction of a disulphide bond between residues 155 and 290, introduction of two ‘cavity-filling' substitutions at positions 190 and 207, and attachment of a heterologous trimerization motif to the C terminus of HRB. These modifications led to the generation of a stable prefusion molecule with modest expression levels, but excellent immunogenicity in mice and macaques^10^.

An RSV F protein that has both high expression levels and maintains a stable prefusion conformation would, therefore, be a promising subunit vaccine candidate against RSV. Thus we sought to identify novel stabilizing mutations based on an understanding of the molecular basis of the prefusion instability and refolding pathway. The high degree of disorder observed previously in the HRA region, and especially in the apex of the protein^10^,^12^, suggested that stabilization of this region might be important to maintain the prefusion conformation. Therefore, we focused on structure-based strategies to stabilize the apex by preventing the refolding of RR1 by arresting the fusion peptide and the hinging of α-helix 4. In addition, we sought to lock the RR2 domain structure in place by reducing the charge repulsion around the top of HRB, which is found at the bottom of the RSV F head. These strategies led to the generation of a highly stable prefusion RSV F that produced in high yields and protected cotton rats against RSV challenge.

## Results

### Design of prefusion-stabilized RSV F

**Figure 1:**
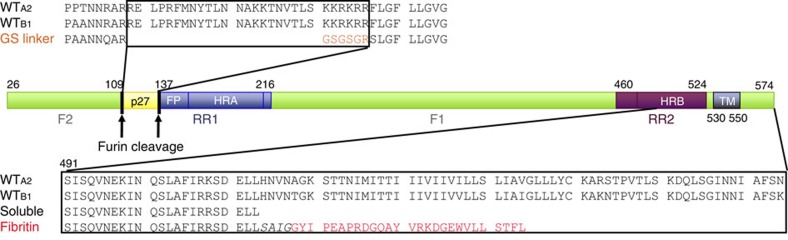
Schematic representation of RSV F_0_ with refolding region 1 (RR1 in blue), refolding region 2 (RR2 in purple), p27 (yellow) and remainder (green). F_0_ protein is cleaved at two positions (arrows) to generate F1 and F2. Upper sequence alignment shows design of single-chain (SC) with short linker region (orange) compared with fragment of Fwt of strain A2 (subgroup A) and B1 (subgroup B). Lower sequence alignment shows design of soluble truncated variant and variant with fibritin domain (red) compared with fragment of Fwt of A2 and B1.

To stabilize the soluble recombinant RSV F ectodomain, several RSV F variants were designed with different modifications. To compensate for the loss of the stabilizing transmembrane region, variants were designed with a heterologous trimerization domain^20^, which proved successful for PIV5 and RSV F^10^,^11^,^16^ (Supplementary Note 1). To prevent the release of the fusion peptide, single-chain (SC) variants were designed with a short linker between the F1 and F2 subunits of RSV F (Fig. 1). SC variants of the B1 strain with a short linker showed higher expression levels and only the SC variant combined with the fibritin domain (SC_B1_) showed higher expression levels and trimeric structure (Supplementary Table 1). The same modifications in the background of the RSV F A2 (SC_A2_) also resulted in higher expression and trimerization (Supplementary Table 1). However, over time the trimeric protein gradually changed into the postfusion conformation as determined by the loss of binding to the prefusion-specific antibody CR9501 (ref. 21).

### Stabilizing the α4–α5 hinge loop

**Figure 2:**
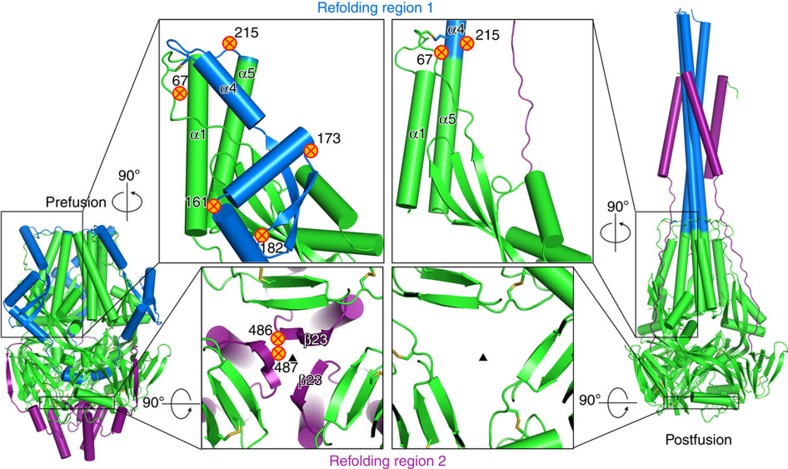
Strategy for prefusion F protein stabilization. RSV F trimer and magnified views of prefusion F (left) and postfusion F protein (right) indicating refolding region 1 (RR1, blue) and refolding region 2 (RR2, purple) and location of amino acid substitutions indicated by crossed circles. The substitutions in the RR1 are designed to prevent the formation of the long helix. The substitutions in the RR2 are designed to reduce the negative charge repulsion between the β23 strands.

**Figure 3:**
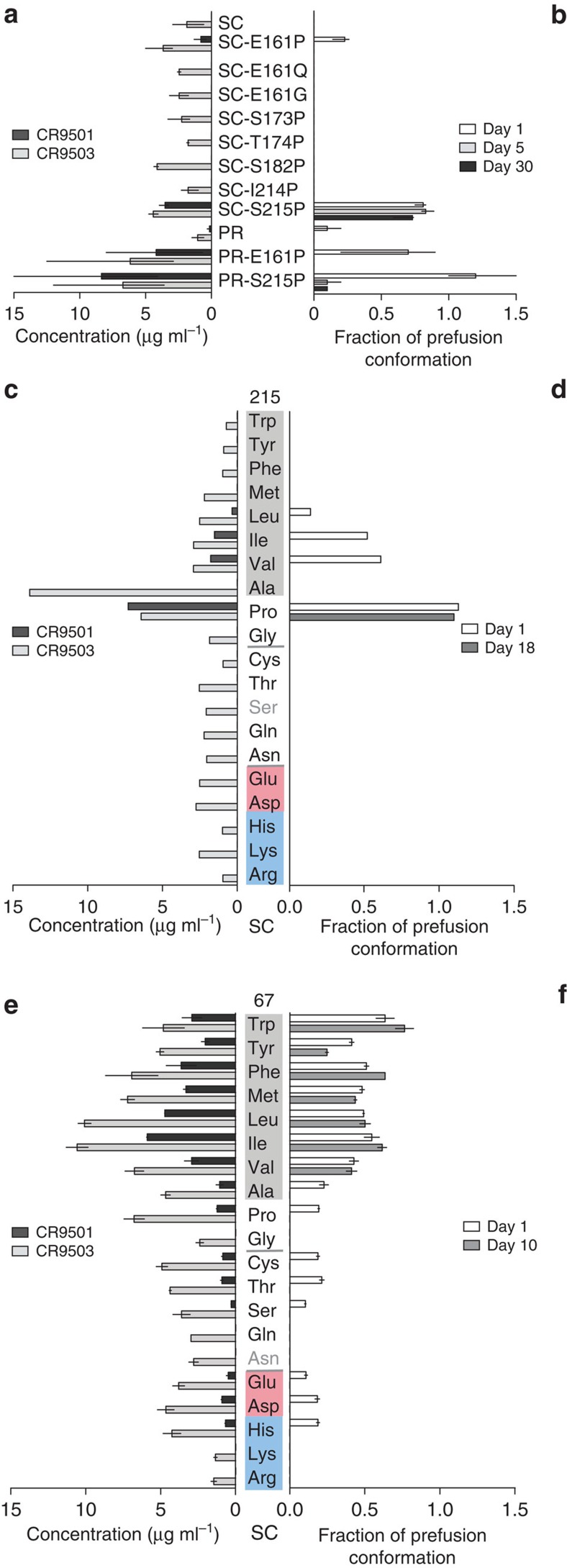
Amino acid substitutions at positions 67 and 215 increase expression level and prefusion stability of the F protein. (**a**,**b**) Protein expression levels and prefusion stability of RSV F SC_A2_ variants and processed variants (PR_A2_) with substitutions in RR1 (*n*=2–4). (**c**,**d**) Protein expression levels and prefusion stability of RSV F SC_A2_ variants with all 20 amino acid substitutions at position 215 (*n*=1) and (**e**,**f**) at position 67 (*n*=2). Protein expression levels in cell culture supernatants were tested 72 h post transfection and fraction of RSV F protein binding to prefusion specific CR9501 antibody on the day of harvest and after storage at 4 °C for indicated period of time. (**a**,**b**,**e** and **f**)—bars represent average of 2–4 measurements, lines represent range of values; (**c**,**d**)—bars represent single measurement. Amino acids are grouped according to physicochemical characteristics (grey: hydrophobic, red: negative charge, blue: positive charge). Variants in this and next figures are based on strain A2.

To preserve the protein in the prefusion conformation, we wanted to prevent the transition to the postfusion conformation by stabilizing the regions between the secondary structural elements that assembled into a long coiled-coil during the transformation from pre- to postfusion RSV F. In an attempt to stabilize the turns between α2–α3, α3–β3, β3–β4 and α4–α5, proline residues were introduced because of their restricted backbone dihedral angles (Fig. 2, Supplementary Fig. 2). Of all single amino acid substitutions at turns, substitution of position 161, 182 and 215 with proline resulted in higher expression levels, and E161P and S215P also increased protein stability (Fig. 3a,b). When the E161P or S215P were introduced in a furin-processed (PR) version of RSV F without the short loop, the expression of both increased more than sixfold and at day 1 the amount of prefusion conformation was ∼70% for PR-E161P and 100% for the PR-S215P variant (Fig. 3a,b). To understand the strong stabilizing effect of S215P, all 20 substitutions at position 215 were made and the variants were tested for expression and stability of prefusion F protein. As shown in Fig. 3c,d, only proline had a major impact on the stability and expression whereas some medium-sized hydrophobic side chains had a minor effect.

### Stabilizing α4 movement

A second strategy to stabilize prefusion F protein was the restriction of the α4 movement. To stabilize the apex and the disordered α4 helix and the β2–α1 loop, the attachment of the β2–α1 loop to the three-helix bundle was optimized (Fig. 2). Because a side chain at position 67 was the most likely candidate to improve the interaction with the three-helix bundle, all substitutions at position 67 were made and the variants were tested for expression and stability of prefusion F protein. As shown in Fig. 3e,f, primarily hydrophobic residues and particularly Ile, Leu and Met at position 67 were able to increase expression and stability.

### Reduction of charge repulsion in RR2

**Figure 4:**
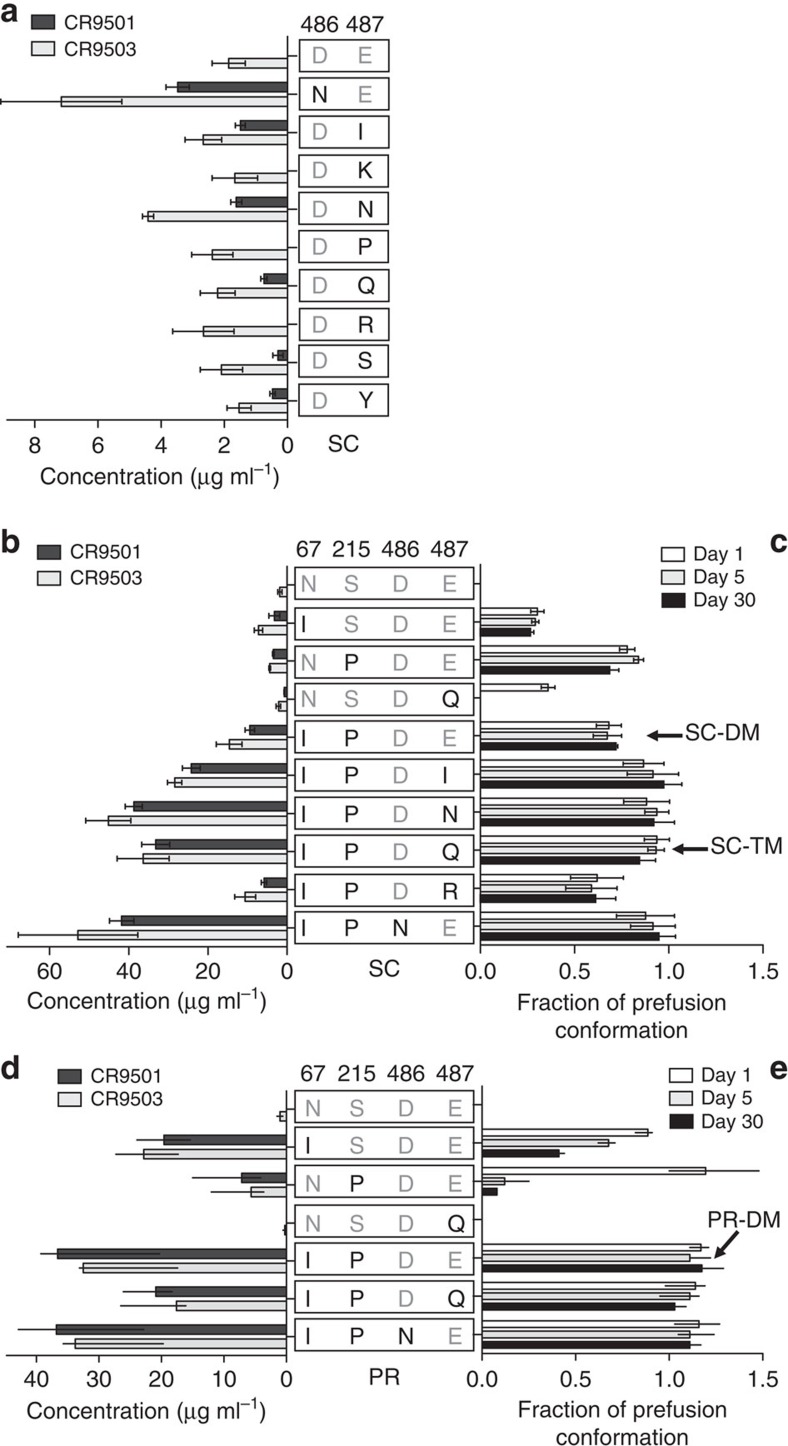
Amino acid substitutions have additive effect on RSV F protein expression and stability. (**a**) Protein expression levels of RSV F SC_A2_ variants with substitutions at position 486 and 487. (**b**,**c**) Protein expression levels and prefusion stability of RSV F SC_A2_ variants with multiple substitutions. (**d**,**e**) Protein expression levels and prefusion stability of processed RSV F PR_A2_ variants with multiple amino acid substitutions. Protein expression levels in cell culture supernatants were tested 72 h post transfection and fraction of RSV F protein binding to prefusion-specific CR9501 antibody on the day of harvest and after storage at 4 °C for indicated period of time. (**a**–**c**) *n*=3–4, Mean±s.e.; (**d**,**e**) bars represent average of 2–4 measurements, lines represent range of values.

A third strategy to stabilize prefusion F protein was the reduction of the negative charge repulsion in RR2 due to clustered negative charges at the top of the HRB. Therefore, an attempt was made to increase the stability of the metastable SC variant by amino acid substitutions of the negatively charged residues at positions 486 and 487 (Fig. 4a). Interestingly, none of the mutants showed a higher prefusion stability beyond day 1 (data not shown), but most showed higher expression levels in comparison to SC.

When the S215P and N67I substitutions were combined in a single construct, a stable prefusion F protein was obtained that had an eightfold higher expression level over the SC variant (Fig. 4b,c). Addition of one of the substitutions at position 486 or 487 increased protein expression levels threefold more. In the processed version of RSV F in which the fusion peptide is not fixed, the additive stabilizing effects of the individual mutations at 67 and 215 were substantial. The N67I substitution had the strongest effect on both the expression level and stability but fully stable prefusion F protein was obtained only when the 67 and 215 substitutions were combined, resulting in a 20-fold expression level increase (Fig. 4d,e).

### Biochemical evaluation of stabilized prefusion RSV F

**Figure 5:**
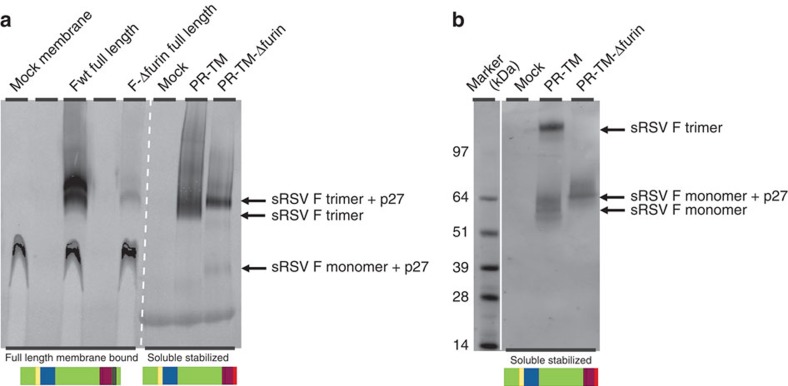
Influence of p27 on trimerization. (**a**, left panel) Full-length RSV F A2 and a variant mutated in one of the furin cleavage sites (F-ΔFurin: R135Q, R136Q) were expressed in HEK293T cells and the purified membrane fractions were analysed by NativePAGE followed by Western blot to evaluate expression and quaternary structure. (**a**, right panel) Soluble, stabilized processed F variant with fibritin motif (PR-TM: N67I, S215P, D486N) and a furin site cleavage mutant (PR-TM-ΔFurin) were analysed on NativePAGE followed by Western blot and (**b**) SDS–PAGE followed by Western blot. For SDS-PAGE, samples were treated with standard 0.1% SDS buffer without reducing agents and without boiling. (bottom) Schematic representations of RSV F with identical colour coding as Fig. 1 with additional transmembrane region (dark green) and fibritin domain (red).

To characterize the prefusion RSV F protein, the double mutant SC variant with the optimized apex (N67I, S215P: SC-DM) and the triple mutant variant with the extra substitution that reduced repulsion in the RR2 domain (N67I, S215P and E487Q: SC-TM) were purified and analysed. The trimeric state of the purified protein was confirmed by SEC-MALS analysis and NativePAGE (Supplementary Fig. 3a–c). The prefusion conformation was confirmed by ELISA (Supplementary Fig. 3d). Negative stain electron microscopy (EM) was conducted on the protein to verify the morphology. A two-dimensional class average showed a compact trimer with a similar morphology to the typical diamond-shaped head of the recently crystallized prefusion RSV F (Supplementary Fig. 3e)^10^,^12^. The heat stability of the purified prefusion protein was determined by differential scanning fluorometry (DSF). Protein refolding from prefusion to postfusion conformation detected using DSF occurred at 52.0 and 57.7 °C for SC-DM and SC-TM, respectively (Supplementary Fig. 3f). Heated proteins no longer bound to the prefusion-specific Mab CR9501 but are still bound to CR9503 (data not shown). Attempts to generate a soluble SC RSV F protein that would include the p27 region by mutation of both furin cleavage sites did not result in detectable F protein (Supplementary Table 1). To understand the impact of p27 on protein folding, a single furin-site knockout was introduced in the native full-length F protein and analysed for expression levels. Again, the furin-site knockout variant showed little expression of F protein compared with the normally processed full-length F protein (Fig. 5a). Therefore, the furin-site knockout was introduced in a soluble F variant stabilized in the prefusion conformation that shows high expression levels. Although the expression level of the furin-site knockout variant was lower, a clear band for RSV F trimer could be detected in NativePAGE (Fig. 5a). Despite the presence of the fibritin trimerization domain, over 6% of the protein that contained the p27 peptide was monomeric, suggesting a possible destabilizing effect of the p27 peptide on trimer formation. Because the fibritin motif in the soluble F protein will drive the trimerization and NativePAGE conditions may not be appropriate to evaluate the destabilizing effect of p27 on trimerization, both proteins were subjected to denaturing conditions to evaluate stability differences of quaternary structure (Fig. 5b). Indeed, incubation with 0.1% SDS at room temperature was able to monomerize 97% of the variant with the attached p27 peptide despite the presence of the fibritin domain, while for the F protein without the p27 peptide, only 50% of the trimer was monomerized suggesting a destabilizing effect of p27 on trimer formation.

**Figure 6:**
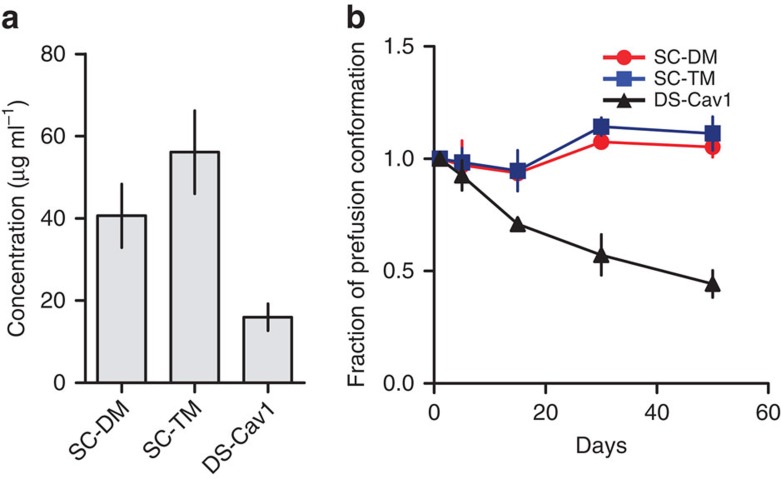
Comparison to DS-Cav1. (**a**) Protein expression levels for double mutant single chain (SC-DM) and triple mutant single chain (SC-TM) were compared with DS-Cav1 (ref. 10) in cell culture supernatants 72 h post transfection and (**b**) the fraction of RSV F protein binding to prefusion-specific CR9501 antibody after storage at 4 °C for indicated days. Bars or symbols represent average from two measurements, lines represent range of values. On (**b**) prefusion F fraction at day of harvest (day 0) is normalized to 1.

Expression levels of SC-DM and SC-TM were compared with DS-Cav1, a prefusion F protein stabilized by cavity-filling mutations and a disulphide bridge^10^. As shown in Fig. 6, the SC-TM variant expressed to almost three times higher levels and both SC-DM and SC-TM remained stable in the culture supernatants for 50 days, whereas DS-Cav1 gradually lost prefusion conformation. Purified SC-TM has remained antigenically stable in the prefusion conformation at 4 °C for more than 450 days based on binding to CR9501 (data not shown).

### Crystallographic analysis

**Figure 7:**
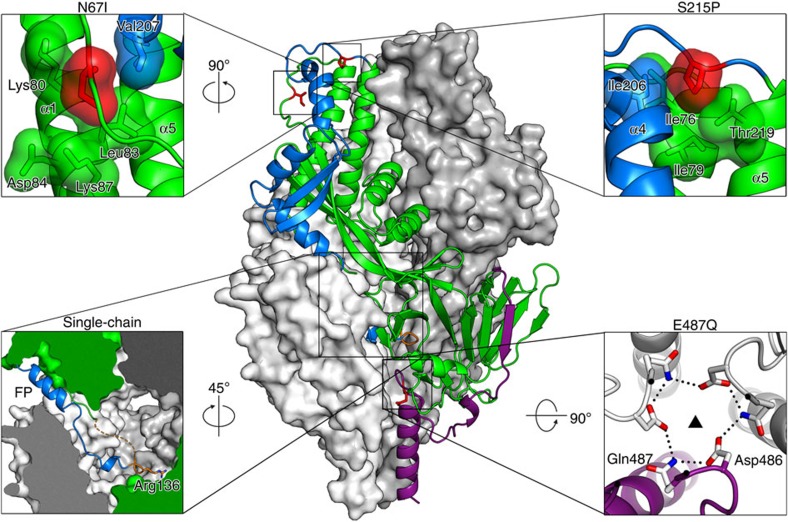
Crystal structure of SC-TM. The trimer is displayed with two protomers as white and grey molecular surfaces and the third protomer as a green ribbon. RR1 with its N-terminal FP is dark blue, and RR2 is dark purple. The stabilizing mutations (Ile67, Pro215 and Gln487) are coloured red (except in the lower right panel), and the loop connecting F1 and F2 is orange. Each of the smaller panels depicts a magnified view of one of the four stabilizing strategies, and their orientation with respect to the main panel is indicated. The lower left panel shows a slice through the trimer, and the dashed orange line represents the disordered portion of the loop. In the lower right panel, hydrogen bonds are depicted as black dotted lines.

We determined the X-ray crystal structures of two prefusion F variants with high expression levels and stability to better understand the effect of these mutations on the prefusion conformation (Fig. 4b-e). Structures of SC-TM (N67I, S215P and E487Q) and the processed variant with the optimized apex PR-DM (N67I, S215P) were determined to 2.4 and 2.3 Å, respectively (Table 1). The structures of SC-TM and PR-DM were very similar to each other, with a root-mean-square deviation of 0.40 Å for 445 Cα atoms (Supplementary Figs 4 and 6). In the SC-TM structure, electron density for parts of the flexible loop that connects F2 and F1 was observed within the central cavity of the trimer. As the C terminus of F2 entered the trimeric cavity, electron density became weaker, and linker residues QARGSG could not be accurately placed due to the disorder. After this gap, density became better ordered, allowing linker residues in the turn (SGRS) to be accurately placed (Fig. 7). In particular, the side chain of the Arg136 residue at the tip of the loop was well ordered and occupied a pocket formed by residues from its own protomer, as well as the fusion peptide of an adjacent protomer. Overall, the conformation adopted by the turn allowed residues in the fusion peptide to remain in the same favourable conformations as those observed in the processed structure (Supplementary Fig. 4).

In both structures the loop between α4 and α5, which includes Pro215, is well ordered with the proline side chain pointing down towards the interface of the triple helix formed by α1, α4 and α5, and into a hydrophobic pocket formed by residues Val76, Ile79, Ile206 and Thr219. The conformation of this region, however, could be influenced by an adjacent trimer in the crystal lattice, which packs head-to-head about the two-fold crystallographic axis. The electron density for Ile67 is weaker than that observed for Pro215, due in part to the intrinsic flexibility of this solvent-exposed, unstructured region of the F2 subunit. Nevertheless, the side chain of Ile67 points towards the interface of α1 and α4, and packs against residues Leu83 and Val207 (Fig. 7). The third stabilizing mutation, E487Q, which was only present in the SC construct, does not result in a substantial change in side chain conformation. However, conformational changes are observed for Phe488 and Asp489, with the side chain of the former adopting a different rotamer in the SC-TM structure. It is unclear as to whether this conformational change is due to the E487Q mutation or to the presence of the SC loop, which packs against the alternate Phe488 side chain.

Both structures presented here were similar to DS-Cav1 (PDB ID: 4MMU), with a root-mean-square deviation of 0.73 and 0.80 Å for PR-DM and SC-TM, respectively. The two regions that differed the most from the DS-Cav1 structure included the loop in F2 containing the N67I substitution and the loop in F1 between α4 and α5 containing the S215P substitution (Supplementary Fig. 4). Thus, the N67I and S215P substitutions preserved the overall conformation of the prefusion state and only introduced small, local differences in two loops.

### Immunogenicity and efficacy of prefusion RSV F protein

To evaluate the prefusion RSV F protein as a vaccine candidate, the immunogenicity and protective efficacy of the SC-DM protein was evaluated in animal models and compared with that of postfusion F protein. Mice were immunized intramuscularly with 0.5 μg of RSV F at weeks 0, 4 and 8 with or without poly(I:C) as an adjuvant. All mice generated antibody titers against the respective immunogen as measured by ELISA (data not shown). The prefusion SC-DM construct induced significantly higher virus neutralization assay (VNA) titers against RSV A2 compared with postfusion F protein. Furthermore, the adjuvanted prefusion F protein construct induced 10-fold higher neutralizing titers than the adjuvanted postfusion RSV F construct (Supplementary Fig. 5a).

**Figure 8:**
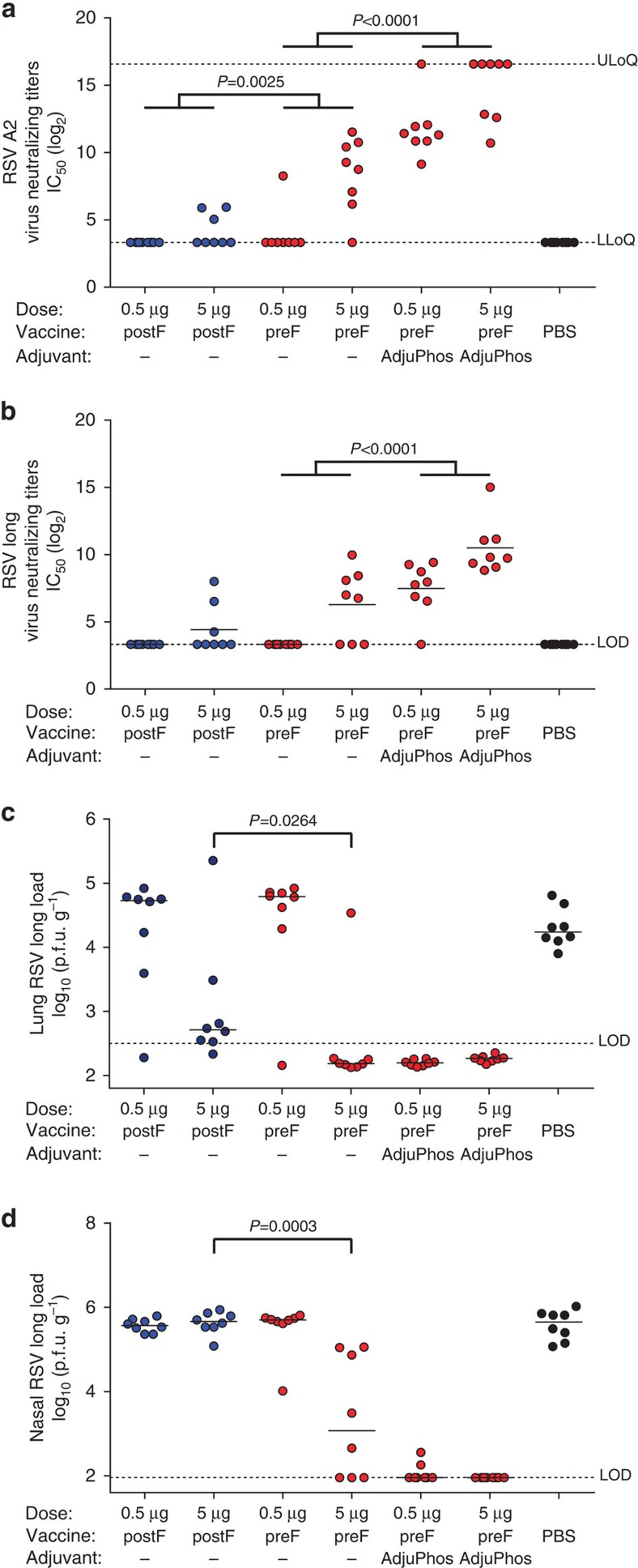
Prefusion RSV F protein is immunogenic and protective in Cotton rats. Female Cotton rats (*n*=8 per group) were immunized IM with 0.5 or 5 μg stabilized prefusion RSV F protein (SC-DM) or RSV F protein in postfusion conformation in a prime—boost regimen at week 0 and week 4. SC-DM groups at the same protein doses was further combined with AdjuPhos. Serum was collected on day 49 and plaque reduction assay was performed using (**a**) RSV A2 and (**b**) Long as described in Materials and Methods. RSV virus titer was determined in (**c**) the lungs or (**d**) nose tissue homogenates of cotton rats at day 5 after challenge (day 54 after prime immunization) as determined by plaque forming units per gram of tissue. Horizontal lines indicate medians. Limit of detection is shown as dotted line. Groups were statistically compared using a non-parametric Cochran-Mantel-Haenszel test. Comparisons across doses are indicated by horizontal lines above the 0.5 and 5 μg groups. Significance was accepted at the *P*<0.05 level and *P*-values below 0.05 are indicated in the figure.

Because the cotton rat model is more permissive to RSV infection than BALB/c mice, we chose this animal model for challenge studies^22^,^23^. Cotton rats were immunized twice with 0.5 or 5 μg of either SC-DM prefusion or postfusion F proteins, both without adjuvant or twice with SC-DM prefusion F protein in combination with AdjuPhos (dosing at weeks 0 and 4). At week 7, animals were bled and RSV neutralizing titers in serum were determined. SC-DM induced a higher neutralization titer than the postfusion RSV F protein against RSV A2, whereas the difference for RSV Long was less pronounced likely due to overall low titers against RSV Long. Addition of AdjuPhos significantly enhanced the neutralizing titers induced by the SC-DM prefusion F protein (Fig. 8a,b).

Cotton rats were challenged intranasally with RSV Long at week 7 and 5 days later RSV viral loads in the lungs and the nose were measured. Immunization with 5 μg prefusion F protein significantly reduced the viral load compared with postfusion F protein. Viral load on challenge was reduced to non-detectable levels in the lungs of seven out of eight animals that had been immunized with the prefusion F protein, whereas only one out of eight animals receiving the postfusion F protein showed non-detectable viral load in this compartment (Fig. 8c). Protection against challenge virus replication in the upper respiratory tract is more difficult to establish and was not achieved in animals immunized with postfusion F protein, irrespective of the protein dose. In contrast, animals that were immunized with 5 μg of prefusion F protein showed a significantly reduced viral load in the nose compared with the postfusion F protein group (Fig. 8d).

The adjuvanted prefusion F protein at a dose of 5 μg gave complete protection in lungs and nose, and even with 0.5 μg of adjuvanted prefusion protein complete protection against challenge virus was observed in the lung and in six out of eight animals in the nose, indicative of a 10-fold dose sparing effect for the adjuvanted protein. Furthermore, the sera showed neutralization against a broad range of subtype A and B strains (Supplementary Fig. 5b).

## Discussion

The RSV F protein exists in a labile, high-energy state that refolds irreversibly to drive membrane fusion. Because RSV F is the main target of neutralizing antibodies, stabilization of the protein in the high-energy prefusion conformation is required to obtain a potent subunit immunogen^5^,^10^. To prevent the assembly of the prefusion RR1 structural elements into one long coiled-coil as happens on transition into the postfusion state, proline residues were introduced in the loops and turns between the secondary structure elements. Although a proline at position 161 and 182 did increase expression levels, only the S215P substitution in the loop between α4 and α5 at the apex had a substantial stabilizing effect. Helix α4 is the first structural element that assembles on top of the structurally conserved, preformed α5 helix base. The flexible α4–α5 loop must then be able to hinge around the nearby conserved cysteine bridge (C69–C212). It is interesting to note that S215P in the crystal structure has interaction with the top of the hydrophobic core formed by helices α1, α4 and α5 at the apex of the protein. When compared with other hydrophobic substitutions at this position, only proline has a strong effect (Fig. 3c,d). Because the hydrophobic residues isoleucine and valine show only a partial stabilizing effect and lower expression, a proline at position 215 has additional benefits and may prevent hinging of α4 by both rigidifying the flexible turn and locking the α4–α5 loop to the hydrophobic core. We note that the S215P in the α4–α5 hinge loop is at a structurally equivalent position as the HIV-1 I559P, which is the most successful mutation to stabilize the HIV-1 trimeric prefusion structure^24^. These equivalent stabilizing mutations suggest that a common early triggering event could exist for class I viral fusion proteins.

The different conformations adopted by the disordered β2–α1 loop and its variable interaction with α1 and α4 (refs 10, 12, 13, 25) suggest that the β2–α1 loop could influence the movement of α4. Thus, improving the interaction with α1 and α4 could stabilize the apex region. Residue 67 in the β2–α1 loop was selected for site-directed mutagenesis because it is most likely less antigenically important than the charged residues at position 65, 66 and 68 and because if all prefusion RSV F structures are compared, the Asn67 side chain does not have a fixed position and is able to reach helix α1 and α4 that form a three-helix bundle with α5. Our results showed that indeed hydrophobic residues and especially isoleucine increased expression levels and had a dramatic impact on the stability by hydrophobic interaction with α1–α4 (Fig. 3e,f). The mutations at position 67 and 215 in the β2–α1 loop and the α4–α5 loop, respectively, provide two independent remedies to prevent helix α4 from moving and hinging. The combination of substitutions N67I and S215P showed a clear additive effect on expression levels and also on stability of prefusion F protein. The high expression and prefusion stability agree with proper folding that has passed cellular quality control.

Although the apex showed the highest level of disorder in previous prefusion RSV F crystal structures^10^, the bottom of the RSV F head is also an area of instability. In this study we tried to stabilize the bottom of the head by reducing the negative charge repulsion at the interface between the protomers. None of the substitutions at position 486 and 487 had a strong stabilizing effect on the semi-stable SC protein but when they were applied to the RSV F variant with the stabilized apex (SC-DM) the mutations at the bottom did show an effect on both heat stability and expression levels (Fig. 4 and Supplementary Fig. 3f). The E487Q substitution reduced the negative charge repulsion and the glutamine could hydrogen bond across the trimer with D486 from another protomer (Fig. 7). Most likely, D486N has a similar structural effect. A previous attempt to increase prefusion F protein stability by neutralizing the negative patch was not successful^10^. All three negative charges were substituted (D486H-E487Q-D489H), resulting in lower yields and a tendency to aggregate along with sensitivity to low pH (ref. 10). This more substantial change would not allow the hydrogen bond network observed with the single E487Q substitution, and the six clustered histidines could cause charge repulsion at low pH.

**Figure 9:**
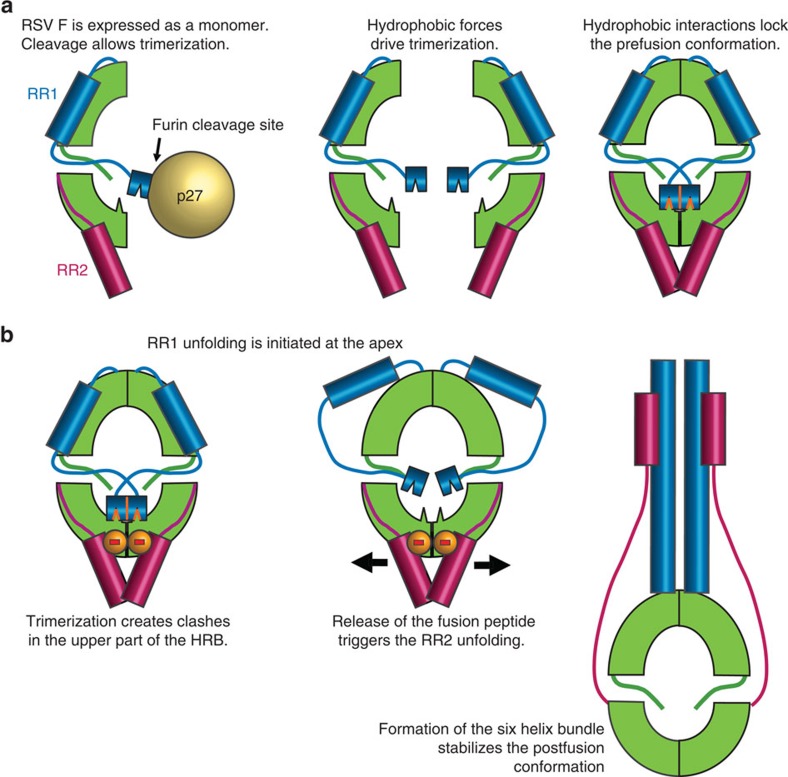
Schematic representation describing how cleavage can activate trimerization and how trimerization causes an inherent instability in the RR2. (**a**) Trimerization of monomeric RSV F is obstructed by the uncleaved bulky glycosylated p27 region. Cleavage of F0 allows trimerization and hydrophobic interaction of the fusion peptide with adjacent protomer. (**b**) The trimerization creates an unstable area in the RR2 through the alignment of a repulsive ring of negative charges contributed by residues Asp486 and Glu487 that are located on β23 that contains an unstable β-bulge (at the edge of β-sheet 3, Fig. 2). The repulsive electrostatic and static forces are countered by the hydrophobic forces linking the FP to the bottom of the cavity. After RR1 is triggered to refold by an unknown mechanism at the apex, it pulls away the stabilizing hydrophobic FP from the cavity which will destabilize the repulsive ring. This results in release of RR2 and ultimately binding of HRB to the HRA coiled-coil to form a 6-helix bundle that is characteristic of the postfusion conformation.

Exploration of the stabilizing strategies for RSV F and crystallographic analysis of the prefusion RSV F proteins offers a putative mechanism of protein refolding from the pre- to postfusion conformation. It is interesting to note that none of the other proline substitutions at the various RR1 hinge points had a stabilizing effect except for the S215P substitution in the α4–α5 loop, which is at the opposite end of the RR1 domain compared with the fusion peptide. Even substitution E161P that was designed to prevent the helix formation by stabilizing the turn and reducing the negative charge repulsion hardly had any stabilizing effect. Another interesting structural observation on HRA is seen in the crystal structure of RSV F containing an introduced disulphide bond between residues 155 and 290 (PDB ID: 4MMQ)^10^. In this structure, a large part of the RR1 region is disordered, although the fusion peptide is fixed in its position by the introduced disulphide bridge that connects it to the β2 strand. This suggests that the release of the fusion peptide from inside the cavity is not required for RR1 refolding and can occur at a later stage. Therefore, the release of the fusion peptide could be triggered by an event (like receptor binding) at the apex that results in the release of α4 and concomitant hinge motion in the α4–α5 loop. This critical hinge will pull on RR1 and eventually pull the fusion peptide from its position like the hinging of a shoulder joint could liberate a hand from a pocket without relying on a movement from the elbow or wrist (Fig. 9).

The X-ray structure of the single chain SC-TM showed that the structure was very similar to processed prefusion structures (Supplementary Fig. 4). Hence, even connected with a linker, the fusion peptide and the C terminus of F2 are located inside the hollow void of the F trimer. Still unknown is how this looped β-hairpin of fusion peptide and C terminus of F2 ended up there. It either tunnelled in through the small pore on the side of the trimer (Figs 7 and 9) or the loop was already there before the pore formed. The largest diameter measured in the pore is about 13 Å, which would be too small to consider the entrance of a tight β-hairpin (fusion peptide and C terminus of F2). Therefore, the observation that the β-hairpin of fusion peptide and the C terminus of F2 is located in the cavity might hint to a state where the fusion peptide and the F2 C terminus were already folded in their current position in monomeric F protein because the β-hairpin is too bulky to tunnel into the interior via the pore of trimeric F protein. Consequently, if this would also apply for wild-type RSV F, the presence of the bulky p27 domain in the folded unprocessed monomer would obstruct full trimer formation. It is only after cleavage that the monomers can assemble and form an unstable native trimer with a trapped F2 C terminus and a fusion peptide that provides some stability at the bottom of the head by hydrophobic interactions with β-sheet 3, a neighbouring fusion peptide and the walls of the cavity. When RSV F variants were compared, it was shown that F variants that could not be processed by furin and still contained the p27 peptide were hardly expressed and it was indeed shown that the p27 peptide had a negative impact on trimerization even when a trimerization motif was present (Fig. 5). Trimerization after cleavage is new in the RSV field, and may also be unique to RSV F since uncleaved flu HA, PIV5 F and HIV-1 Env have all been shown to exist in trimeric conformations that are very similar to the cleaved protein. The trimerization of RSV F also brings some inherent instability exemplified by the unfavourable β-bulge in β23 and alignment of a series of negative charges in a repulsive ring around the three-fold axis which causes electrostatic repulsion at the trimer interface (Figs 2 and 9). Therefore, when the fusion peptide is released from the cavity after RR1 C-terminal refolding, the stabilization by hydrophobic interactions will vanish and the repulsion at the trimer interface will prevail and trigger the refolding of the RR2 domain (Fig. 9).

The structural insights and stabilization strategies presented here produced a prefusion RSV F protein that was stable on storage at 4 °C for more than a year and was also heat stable (Supplementary Fig. 3), which is an important requirement for vaccine development. As expected from a stabilized prefusion RSV F protein, higher titers of neutralizing antibodies were induced in rodents compared with postfusion RSV F. Especially the efficacy of the prefusion protein to protect cotton rats against replication of RSV challenge virus in the nose was significantly higher than the efficacy of the postfusion F. Even a prime boost with non-adjuvanted prefusion protein almost completely protected against RSV replication in the nose. With AdjuPhos as adjuvant protection could be reached with a prime boost of 0.5 μg of prefusion protein.

In conclusion, with a minimal number of unique amino acid substitutions focused on stabilization of helix α4 in the disordered apex, a stable prefusion RSV F protein was constructed that could be produced to high yield in mammalian cells. Prefusion RSV F elicited broadly neutralizing antibodies and induced full protection against viral challenge in cotton rats. Protein engineering for vaccine development has led to a stabilization strategy that has brought new insights in the unique role of the enigmatic p27 peptide in RSV F function and additional broader insights in triggering events in class I viral fusion proteins.

## Methods

### Ethics statement

This study was carried out in strict accordance with the recommendations in the Institutional Animal Care and Use Committee guidelines for cotton rat studies. Studies in mice were performed according to Dutch law (Dutch Animal Experimentation Act) and Guidelines on the Protection of Experimental Animals by the Council of the European Committee (EU Dir. 86/609) after approval by the Dier Experimenten Commissie (permit number 21300). All invasive procedures were performed under isofluorane anaesthesia, and all efforts were made to minimize animal suffering.

### Viruses and cells

The following RSV strains were used: A2 (ATCC, VR1540), Long (ATCC, VR-26), B1 (ATCC, VR2542 ), RSV 05-036549 (kindly provided by Dr F. Coenjaerts, Utrecht Medical Centre, Utrecht, The Netherlands), RSV A CL57, RSV A CL25, RSV B 423, RSV B CL19 (kindly provided by Dr D. Roymans, Janssen Pharmaceuticals, Beerse, Belgium) and RSV B15/97 (kindly provided by Sigmovir Biosystems, USA). Large-scale RSV preparations were produced by infecting Hep2 cells (ATCC, CCL-23), and harvesting the cell culture supernatants 3–4 days after infection. Cell culture supernatants were stabilized in 25% sucrose in phosphate-buffered saline (PBS) and stored at −80 °C.

### Expression and purification of antibodies and Fab fragments

Fully human IgG1 anti-RSV F protein antibodies CR9501, CR9503, CR9515 and CR13039 were constructed by cloning the heavy (VH) and light (VL) chain variable regions into a single IgG1 expression vector. PER.C6 cells (Crucell) were transfected with the IgG1 expression constructs and the expressed antibodies were purified from culture supernatants using POROS Mabcapture A chromatography (Applied Biosystems) and then buffer exchanged to 50 mM NaAc, 50 mM NaCl, pH 5.5. Antibody concentration was measured by optical absorption at 280 nm. Antibody quality was also confirmed by size-exclusion chromatography (SEC), SDS–PAGE and isoelectric focusing. CR9501 comprises VH and VL regions of 58C5, which binds specifically to RSV F protein in its prefusion conformation and not to the postfusion conformation^21^. CR9503 comprises VH and VL regions of motavizumab^26^. CR9515 comprises VH and VL regions of D25 antibody^27^. CR13039 comprises VH and VL regions of MPE8 antibody^28^.

### Expression and purification of RSV F protein

Expression plasmids encoding the recombinant prefusion RSV F protein were generated as follows. Parental construct based on RSV A2 (Genbank ACO83301.1) was ordered from GeneArt (Life Technologies). It consists of residues 1–513 of the F protein and fibritin trimerization domain. Amino acid substitutions were introduced by site-directed mutagenesis (QuickChange II Site-Directed Mutagenesis kit, Agilen Biotechnologies). For RSV F in postfusion conformation a DNA construct encoding RSV F residues 1–136 and 146–524 (corresponding to the F protein ectodomain without the fusion peptide)^13^ was synthesized (GeneArt). Recombinant proteins were expressed in 293 Freestyle cells (Life Technologies). The cells were transiently transfected using 293Fectin (Life Technologies) according to the manufacturer's instructions and cultured in a shaking incubator at 37 °C and 10% CO_2_. The culture supernatants containing F protein were harvested on 5th day after transfection. Sterile-filtered supernatants were stored at 4 °C until use. The recombinant polypeptides were purified by a two-step protocol applying a cation exchange chromatography followed by SEC. For the ion-exchange step the culture supernatant was diluted with two volumes of 50 mM NaOAc, pH 5.0, and passed over a 5 ml HiTrap Capto S (GE Healthcare) column at 5 ml min^−1^. Subsequently the column was washed with 10 column volumes of 20 mM NaOAc, 50 mM NaCl, 0.01% (v/v) Tween20, pH 5, and eluted with 2 column volume of 20 mM NaOAc, 1 M NaCl, 0.01% (v/v) Tween20, pH 5. The eluate was concentrated and the protein was further purified on a Superdex200 column (GE Healthcare) using 40 mM Tris buffer, 500 mM NaCl, 0.01% (v/v) Tween20, and pH 7.4 as running buffer. A reduced SDS–PAGE analysis was used to determine purity of the final protein preparation; only protein with purity >95% was used for further analysis. The identity of the band was verified using western blot with CR9503.

### SDS–PAGE analysis and western blot

Cell culture supernatants or purified protein samples were analysed on 4–12% (w/v) Bis–Tris NuPAGE gels, 1 × MOPS (Life Technologies) under reducing or non-reducing conditions and blotted using the iBlot technology (Life Technologies). All procedures were performed according to manufacturer's instructions. For purity analysis the gels were stained with Krypton Infrared Protein Stain (Thermo Scientific) or SYPRO Rubi protein stain (Bio-Rad). The blots were probed with CR9503 at 1 μg ml^−1^, followed by IRDye800CW conjugated anti-human IgG (rabbit; Rockland Immunochemicals, Gilbertsville, PA, USA). The gels and the blot membranes were scanned on an Odyssey instrument (Li-Cor) and images analysed using Odyssey 3.0 software (Li-Cor).

### Confirmation of trimeric state

Multimeric state of the prefusion RSV F protein, purified or in crude cell culture supernatant, was analysed in a NativePAGE Bis–Tris gel system (Life Technologies). For confirmation of the bands identity and for analysis of proteins in crude cell culture supernatants, the gels were blotted as described above. The gels and the blots were analysed on Odyssey instrument as described above. Size of the proteins was estimated in comparison with a Native Standard (NativeMark, Life Technologies).

RSV F protein was monitored by SEC and multi-angle light scattering analysis using a high-performance liquid chromatography system (Agilent Technologies) and miniDAWN TREOS (Wyatt) instrument coupled to a Optilab T-rEX Refractive Index Detector (Wyatt). In total, 40 μg of protein was applied to a TSK-Gel G3000SWxl column (Tosoh Bioscience) equilibrated in running buffer (150 mM NaPi, 50 mM NaCl, pH 7.0). The data were analysed by the Astra 6 software package and molecular weight calculations were derived from the refractive index signal.

### Quantitative octet assay

To measure the concentration of the prefusion RSV F protein in cell culture supernatants, a quantitative Octet-based method was used. The CR9501 and CR9503 antibodies were biotinylated and immobilized at 5 μg ml^−1^ on Streptavidin biosensors (Pall). The antibody-coated biosensors were blocked in mock cell culture supernatant. Quantitative experiment was performed on an OctetRED384 instrument (Pall) at 30 °C shaking speed 1,000 r.p.m. for 300 s. Concentration of the protein was calculated using standard curves, which were prepared for each coated antibody using the SCDM protein, diluted in mock cell culture supernatant. The data analysis was performed using the ForteBio Data Analysis 6.4 software (ForteBio). Negative control samples (mock cell culture supernatants) were used for reference subtraction. The algorithm used was ‘Linear point-to-point'.

### Stability studies

Ability of the prefusion protein to spontaneously convert into postfusion conformation was assessed in a storage stability assay. The crude cell culture supernatant samples were stored at 4 °C and concentration of the F protein in the samples was measured on Octet instrument by quantitative assay as described above. The measurement was done on the day of supernatant harvest (day 1) and after storage for indicated period of time. CR9503 was used to measure the total F protein concentration; CR9501 was used to measure prefusion RSV F protein concentration.

Temperature stability of the purified proteins was determined by DSF. The purified prefusion F protein was mixed with SYPRO orange fluorescent dye (Life Technologies S6650) in a 96-well optical quantitative PCR plate. The optimal dye and protein concentration was determined experimentally (data not shown). All protein dilutions were performed in PBS, and a negative control sample containing the dye only was used as a reference subtraction. The measurement was performed in a quantitative PCR instrument (Applied Biosystems ViiA 7) using the following parameters: a temperature ramp from 25 to 95 °C with a rate of 0.015 °C sec^−1^. Data were collected continuously. The melting curves were plotted using GraphPad PRISM software (version 5.04). Melting temperatures were calculated at the 50% maximum of fluorescence using a non-linear EC50 shift equation.

### ELISA

Binding of the prefusion RSV F proteins to prefusion-specific neutralizing antibodies was tested in an ELISA. The MaxiSorp polystyrene 96-well plates (NUNC) were coated with anti-RSV F monoclonal antibody (MAB8262, Millipore) over-night at 4 °C in PBS. The antibodies were diluted in PBS at 1 μg ml^−1^. The next day, plates were washed with washing buffer (PBS, 0.05% Tween) and blocked in PBS with 1% bovine serum albmin. All incubations were performed at room temperature for 1 h. After each step, plates were washed three times with the Wash buffer. Titrations of the purified RSV F protein were prepared in the washing buffer with 1% bovine serum albmin. Test antibodies were biotinylated according to standard procedure and diluted in the same buffer at 0.25 μg ml^−1^. For detection, Streptavidin-HRP (BD Bioscience) was used at 1:1,000 dilution with a TMB Microwell Peroxidase Substrate System (KLP).

### Electron microscopy

Electron microscopy studies were performed by NanoImaging Services, Inc. (CA, USA). Samples were prepared using continuous carbon grid method. Grids were nitrocellulose supported 400-mesh copper. Samples were prepared by applying 3 μl of sample suspension to a cleaned grid, blotting away with filter paper, and immediately staining with uranyl formate. Electron microscopy was performed using an FEI Tecnai T12 electron microscope, operating at 120 keV equipped with an FEI Eagle 4 k × 4 k CCD camera. Negative stain grids were transferred into the electron microscope using a room temperature stage. Images of each grid were acquired at multiple scales to assess the overall distribution of the specimen. After identifying, potentially, suitable target areas for imaging at lower magnifications, pairs of high magnification images were acquired at nominal magnifications of 110,000 × (0.10 nm per pixel), 67,000 × (0.16 nm per pixel) and 52,000 × (0.21 nm per pixel). The images were acquired at a nominal underfocus of −2 μm (110,000 × ), −3 to −2 μm (67,000 × ) and −4 to −3 μm (52,000 × ) and electron doses of ∼25–45 eÅ^−2^. A two-dimensional class averaging was applied to the images. Hereto, individual particles in the 67,000 × or 110,000 × high magnification images were selected using automated picking protocols^29^. An initial round of alignments was performed and from that alignment class averages that appeared to contain real particles were selected for additional rounds of alignment. A reference-free alignment strategy based on the X-windows-based microscopy image processing package (Xmipp)^30^ was applied, which contains an algorithm that aligns the selected particles and sorts them into self-similar groups of classes.

### Crystallization and structure determination

Crystals were grown by the vapor-diffusion method in hanging drops at 18 °C by mixing prefusion RSV F proteins at 4–5 mg ml^−1^ with a reservoir solution consisting of 1.24–1.34 M K/Na tartrate, 0.2 M Li_2_SO_4,_ and 0.1 M CHES pH 9.5. Prior to freezing in liquid nitrogen, crystals were transferred to a 3.5 M (NH_4_)_2_SO_4_ solution. Initial X-ray diffraction data were collected remotely at the National Synchrotron Light Source beamline X6A, and the final X-ray diffraction data were collected remotely at a wavelength of 0.9792 Å at the Advanced Photon Source beamline SBC 19ID. Data were processed with iMOSFLM^31^ and AIMLESS^32^, and a molecular replacement solution was obtained by PHASER^33^ using the structure of RSV F Cav1 (PDB ID: 4MMR)^10^ as a search model. Iterative rounds of model building and refinement were performed with COOT^34^ and PHENIX^35^, respectively. Final data collection and refinement statistics are presented in Table 1. Ramachandran statistics for the SC-TM and the PR-DM are 96.0% and 97.5% favoured, 3.8% and 2.0% allowed and 0.2% and 0.5% outliers, respectively.

### Mouse studies

Six to eight-week-old specific pathogen-free female BALB/c mice were purchased from Charles River and kept at the institutional animal facility under specific pathogen-free conditions. Animals were randomly distributed in experimental groups of nine animals and were vaccinated intramuscularly in the gastrocnemius of both hind legs (50 μl per leg) with 0.5 μg of F protein with 50 μg of poly(I:C) or 0.5 μg of F protein without adjuvant. All animals received two injections 4 weeks apart. Serum was obtained by submandibular bleeding or heart puncture under isofluorane anaesthesia at weeks 0, 4, 6 and 12.

### Cotton rat studies

Groups of eight female cotton rats between 6–8 weeks of age (Sigmovir Biosystems, Inc., Rockville MD) were immunized intramuscularly with 0.5 or 5 μg RSV F with or without AdjuPhos and boosted 28 days later with the same dose. Animals were challenged at day 49 with RSV A2 with 10^5^ plaque forming units (p.f.u.) and euthanized at day 54. There was a group of cotton rats that was injected with buffer only and served as negative control. Serum samples were collected before prime immunization (day 0), before the boost immunization (day 28) and before challenge (day 49) for VNA titer measurement by a plaque reduction assay. Cotton rats were euthanized 5 days after infection, a time point at which RSV challenge virus reaches peak titers^36^. Lung and nasal tissue were isolated and homogenized. RSV load in the tissue homogenates was determined by virus plaque titration^36^,^37^and presented as p.f.u. per gram of tissue.

### Virus neutralization assay

VNA in mouse serum was determined by a microneutralization assay. RSV-susceptible VERO cells (obtained via Berna Biotech, Bern) were seeded in 96-well cell-culture plates 1 day prior to infection. On the day of infection, serially diluted sera and controls were mixed with 1,000 p.f.u. of RSV in 96-well U-bottom plates and incubated for 1 h at 37 °C. Subsequently, virus/antibody mixes were transferred to 96-well plates containing VERO cell monolayers and transferred to an incubator at 37 °C. Three days later monolayers were washed and fixed with 80% ice-cold acetone. RSV replication was determined by F protein expression. Fixed monolayers were incubated with a biotin-conjugated anti-F monoclonal antibody and washed. Streptavidin-horseradish peroxidase (SA-HRPO) was added to the wells. The wells were washed again, and turnover of the 3,3′,5,5′-tetramethylbenzidine substrate was measured at an optical density of 450 nm (OD450). VNA titers were calculated as the antibody concentration that caused a 50% reduction in the OD450, expressed as IC50 titers.

VNA for cotton rat serum was determined by a plaque reduction assay. In brief, heat-inactivated serum samples were diluted 1:10 with Eagle's minimal essential medium (Lonza) and serially diluted further 1:4. Diluted serum samples were incubated with RSV A2 or A/Long (25–50 p.f.u.) for 1 h at room temperature and inoculated in duplicates onto confluent HEp-2 monolayers in 24-well plates. After 1 h incubation at 37 °C in a 5% CO_2_ incubator, cells were overlayed with 0.75% methylcellulose medium (Sigma) and plates were incubated at 37 °C in the incubator. Four days later the overlay was removed and the cells were fixed with 0.1% crystal violet stain for 1 h, then rinsed and air dried. VNA titers were expressed as IC50 titers, the antibody concentration that caused a 50% reduction in the average plaque count of virus-only incubated cells.

### Statistical analysis

The results of the VNA titer were log-transformed and comparisons of prefusion F and postfusion F protein groups (across dose) or between adjuvanted and non-adjuvanted groups were performed using a non-parametric Cochran-Mantel-Haenszel test. Comparisons between groups containing values below the lower limit of quantification were analysed using censored regression models. Wilcoxon Rank-Sum test with Bonferroni correction for multiple testing was applied when comparing lung titers and nose titers of the postfusion and prefusion groups, omitting the adjuvanted groups and the PBS group. The overall level of significance (alpha) was set at 5%. All statistical analyses were performed in SAS version 9.4.

## Additional information

**How to cite this article:** Krarup, A. *et al*. A highly stable prefusion RSV F vaccine derived from structural analysis of the fusion mechanism. *Nat. Commun*. 6:8143 doi: 10.1038/ncomms9143 (2015).

## Supplementary Material

Supplementary Information Supplementary Figures 1-6, Supplementary Table 1 and Supplementary Note 1

## Figures and Tables

**Table 1:** Data collection and refinement statistics.

	SC-TM	PR-DM
*Data collection*		
Space group	*P*4_1_32	*P*4_1_32
Cell dimensions		
*a, b*, *c* (Å)	168.2, 168.2, 168.2	167.8, 167.8, 167.8
α, β, γ (°)	90.0, 90.0, 90.0	90.0, 90.0, 90.0
Resolution (Å)	45.0–2.4 (2.49–2.40)	46.5–2.3 (2.38–2.30)
*R*_merge_	0.142 (1.327)	0.133 (1.554)
*I/σI*	12.6 (1.9)	14.0 (1.8)
CC(1/2)	(0.64)	(0.63)
Completeness (%)	99.7 (100.0)	100.0 (100.0)
Redundancy	10.5 (10.7)	11.6 (12.0)
		
*Refinement*		
Resolution (Å)	45.0–2.4 (2.47–2.40)	46.5–2.3 (2.36–2.30)
No. of reflections	32,193 (2,647)	36,384 (2,741)
*R*_work_/*R*_free_ (%)	17.8/21.1	18.0/21.7
No. of atoms		
Protein	3,517	3,459
Ligand/ion	35	66
Water	161	173
*B*-factors		
Protein	56.3	56.1
Ligand/ion	58.4	105.7
Water	46.8	48.0
r.m.s. deviations		
Bond lengths (Å)	0.003	0.005
Bond angles (°)	0.70	0.86

PR-DM, furin-processed double mutant; r.m.s., root-mean-square deviation; SC-TM, triple mutant single chain variant.

## References

[b1] Nair H. . Global burden of acute lower respiratory infections due to respiratory syncytial virus in young children: a systematic review and meta-analysis. Lancet 375, 1545–1555 (2010). 20399493 10.1016/S0140-6736(10)60206-1 PMC2864404

[b2] Hall C. B. . The burden of respiratory syncytial virus infection in young children. N. Engl. J. Med. 360, 588–598 (2009). 19196675 10.1056/NEJMoa0804877 PMC4829966

[b3] Glezen W. P., Taber L. H., Frank A. L. & Kasel J. A. Risk of primary infection and reinfection with respiratory syncytial virus. Am. J. Dis. Child. 140, 543–546 (1986). 3706232 10.1001/archpedi.1986.02140200053026

[b4] Zhou H. . Hospitalizations associated with influenza and respiratory syncytial virus in the United States, 1993-2008. Clin. Infect. Dis. 54, 1427–1436 (2012). 22495079 10.1093/cid/cis211 PMC3334364

[b5] Magro M. . Neutralizing antibodies against the preactive form of respiratory syncytial virus fusion protein offer unique possibilities for clinical intervention. Proc. Natl Acad. Sci. USA 109, 3089–3094 (2012). 22323598 10.1073/pnas.1115941109 PMC3286924

[b6] Olmsted R. A. . Expression of the F glycoprotein of respiratory syncytial virus by a recombinant vaccinia virus: comparison of the individual contributions of the F and G glycoproteins to host immunity. Proc. Natl Acad. Sci. USA 83, 7462–7466 (1986). 3532115 10.1073/pnas.83.19.7462 PMC386738

[b7] Smith G. . Respiratory syncytial virus fusion glycoprotein expressed in insect cells form protein nanoparticles that induce protective immunity in cotton rats. PLoS ONE 7, e50852 (2012). 23226404 10.1371/journal.pone.0050852 PMC3511306

[b8] Johnson S. . Development of a humanized monoclonal antibody (MEDI-493) with potent *in vitro* and *in vivo* activity against respiratory syncytial virus. J. Infect. Dis. 176, 1215–1224 (1997). 9359721 10.1086/514115

[b9] Collins P. L. & Melero J. A. Progress in understanding and controlling respiratory syncytial virus: still crazy after all these years. Virus. Res. 162, 80–99 (2011). 21963675 10.1016/j.virusres.2011.09.020 PMC3221877

[b10] McLellan J. S. . Structure-based design of a fusion glycoprotein vaccine for respiratory syncytial virus. Science 342, 592–598 (2013). 24179220 10.1126/science.1243283 PMC4461862

[b11] McLellan J. S. . Structural basis of respiratory syncytial virus neutralization by motavizumab. Nat. Struct. Mol. Biol. 17, 248–250 (2010). 20098425 10.1038/nsmb.1723 PMC3050594

[b12] McLellan J. S. . Structure of RSV fusion glycoprotein trimer bound to a prefusion-specific neutralizing antibody. Science 340, 1113–1117 (2013). 23618766 10.1126/science.1234914 PMC4459498

[b13] Swanson K. A. . Structural basis for immunization with postfusion respiratory syncytial virus fusion F glycoprotein (RSV F) to elicit high neutralizing antibody titers. Proc. Natl Acad. Sci. USA 108, 9619–9624 (2011). 21586636 10.1073/pnas.1106536108 PMC3111287

[b14] Swanson K. . Structure of the Newcastle disease virus F protein in the post-fusion conformation. Virology 402, 372–379 (2010). 20439109 10.1016/j.virol.2010.03.050 PMC2877518

[b15] Yin H. S., Paterson R. G., Wen X., Lamb R. A. & Jardetzky T. S. Structure of the uncleaved ectodomain of the paramyxovirus (hPIV3) fusion protein. Proc. Natl Acad. Sci. USA 102, 9288–9293 (2005). 15964978 10.1073/pnas.0503989102 PMC1151655

[b16] Yin H. S., Wen X., Paterson R. G., Lamb R. A. & Jardetzky T. S. Structure of the parainfluenza virus 5F protein in its metastable, prefusion conformation. Nature 439, 38–44 (2006). 16397490 10.1038/nature04322 PMC7095149

[b17] Earp L. J., Delos S. E., Park H. E. & White J. M. The many mechanisms of viral membrane fusion proteins. Curr. Top. Microbiol. Immunol. 285, 25–66 (2005). 15609500 10.1007/3-540-26764-6_2 PMC7122167

[b18] Jardetzky T. S. & Lamb R. A. Activation of paramyxovirus membrane fusion and virus entry. Curr. Opin. Virol. 5, 24–33 (2014). 24530984 10.1016/j.coviro.2014.01.005 PMC4028362

[b19] Liljeroos L., Krzyzaniak M. A., Helenius A. & Butcher S. J. Architecture of respiratory syncytial virus revealed by electron cryotomography. Proc. Natl Acad. Sci. USA 110, 11133–11138 (2013). 23776214 10.1073/pnas.1309070110 PMC3703984

[b20] Miroshnikov K. A. . Engineering trimeric fibrous proteins based on bacteriophage T4 adhesins. Protein Eng. 11, 329–332 (1998). 9680195 10.1093/protein/11.4.329

[b21] Williamson R. A., Wadia J., Pascual G. & Keogh E. Anti-human respiratory syncytial virus (RSV) antibodies and methods of use. U.S. Patent 2014/0363427 (2014).

[b22] Niewiesk S. & Prince G. Diversifying animal models: the use of hispid cotton rats (Sigmodon hispidus) in infectious diseases. Lab. Anim. 36, 357–372 (2002). 12396279 10.1258/002367702320389026

[b23] Boukhvalova M. S. & Blanco J. C. The cotton rat Sigmodon hispidus model of respiratory syncytial virus infection. Curr. Top. Microbiol. Immunol. 372, 347–358 (2013). 24362698 10.1007/978-3-642-38919-1_17

[b24] Sanders R. W. . Stabilization of the soluble, cleaved, trimeric form of the envelope glycoprotein complex of human immunodeficiency virus type 1. J. Virol. 76, 8875–8889 (2002). 12163607 10.1128/JVI.76.17.8875-8889.2002 PMC136973

[b25] McLellan J. S. . Structure of a major antigenic site on the respiratory syncytial virus fusion glycoprotein in complex with neutralizing antibody 101F. J. Virol. 84, 12236–12244 (2010). 20881049 10.1128/JVI.01579-10 PMC2976384

[b26] Wu H. . Development of motavizumab, an ultra-potent antibody for the prevention of respiratory syncytial virus infection in the upper and lower respiratory tract. J. Mol. Biol. 368, 652–665 (2007). 17362988 10.1016/j.jmb.2007.02.024

[b27] Kwakkenbos M. J. . Generation of stable monoclonal antibody-producing B cell receptor-positive human memory B cells by genetic programming. Nat. Med. 16, 123–128 (2010). 20023635 10.1038/nm.2071 PMC2861345

[b28] Corti D. . Cross-neutralization of four paramyxoviruses by a human monoclonal antibody. Nature 501, 439–443 (2013). 23955151 10.1038/nature12442

[b29] Lander G. C. . Appion: an integrated, database-driven pipeline to facilitate EM image processing. J. Struct. Biol. 166, 95–102 (2009). 19263523 10.1016/j.jsb.2009.01.002 PMC2775544

[b30] Sorzano C. O. . XMIPP: a new generation of an open-source image processing package for electron microscopy. J. Struct. Biol. 148, 194–204 (2004). 15477099 10.1016/j.jsb.2004.06.006

[b31] Battye T. G., Kontogiannis L., Johnson O., Powell H. R. & Leslie A. G. iMOSFLM: a new graphical interface for diffraction-image processing with MOSFLM. Acta. Crystallogr. D Biol. Crystallogr. 67, 271–281 (2011). 21460445 10.1107/S0907444910048675 PMC3069742

[b32] Evans P. R. & Murshudov G. N. How good are my data and what is the resolution? Acta. Crystallogr. D Biol. Crystallogr. 69, 1204–1214 (2013). 23793146 10.1107/S0907444913000061 PMC3689523

[b33] McCoy A. J. . Phaser crystallographic software. J. Appl. Crystallogr. 40, 658–674 (2007). 19461840 10.1107/S0021889807021206 PMC2483472

[b34] Emsley P., Lohkamp B., Scott W. G. & Cowtan K. Features and development of Coot. Acta. Crystallogr. D Biol. Crystallogr. 66, 486–501 (2010). 20383002 10.1107/S0907444910007493 PMC2852313

[b35] Adams P. D. . PHENIX: a comprehensive Python-based system for macromolecular structure solution. Acta. Crystallogr. D Biol. Crystallogr. 66, 213–221 (2010). 20124702 10.1107/S0907444909052925 PMC2815670

[b36] Prince G. A., Prieels J. P., Slaoui M. & Porter D. D. Pulmonary lesions in primary respiratory syncytial virus infection, reinfection, and vaccine-enhanced disease in the cotton rat (Sigmodon hispidus). Lab. Invest. 79, 1385–1392 (1999). 10576209

[b37] Prince G. A., Jenson A. B., Horswood R. L., Camargo E. & Chanock R. M. The pathogenesis of respiratory syncytial virus infection in cotton rats. Am. J. Pathol. 93, 771–791 (1978). 362946 PMC2018360

