# Supplementary material for: A highly stable prefusion RSV F vaccine derived from structural analysis of the fusion mechanism
Source: Nat Commun. 2015 Sep 3;6:8143. doi: 10.1038/ncomms9143 (PMC4569726; doi:10.1038/ncomms9143)
Supplement: Supplementary Information — Supplementary Figures 1-6, Supplementary Table 1 and Supplementary Note 1 [file ncomms9143-s1.pdf]

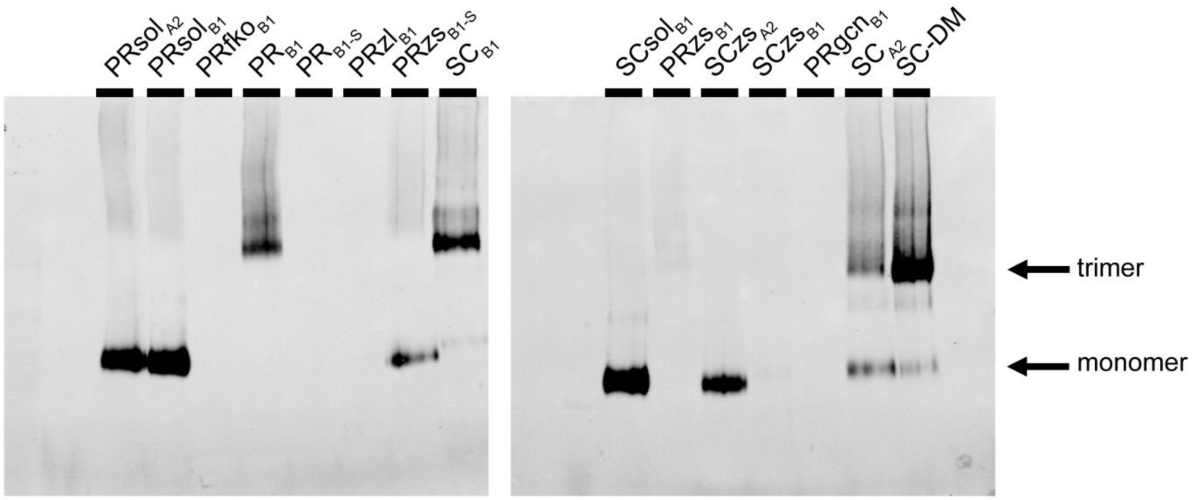

Supplementary Figure 1.

Assessment of quaternary structure of soluble RSV F proteins. Soluble variants of F proteins from A2 and B1 RSV strains were expressed in HEK293 cells. The cell culture supernatants were analyzed on NativePAGE followed by Western blot with RSV-F specific antibody. The bands of 2 different sizes were visible, approximately corresponding to a molecular mass of a fusion protein monomer or trimer (indicated by arrows). More details on the different constructs can be found in **Supplementary Table 1** and in **Supplementary Note 1**.

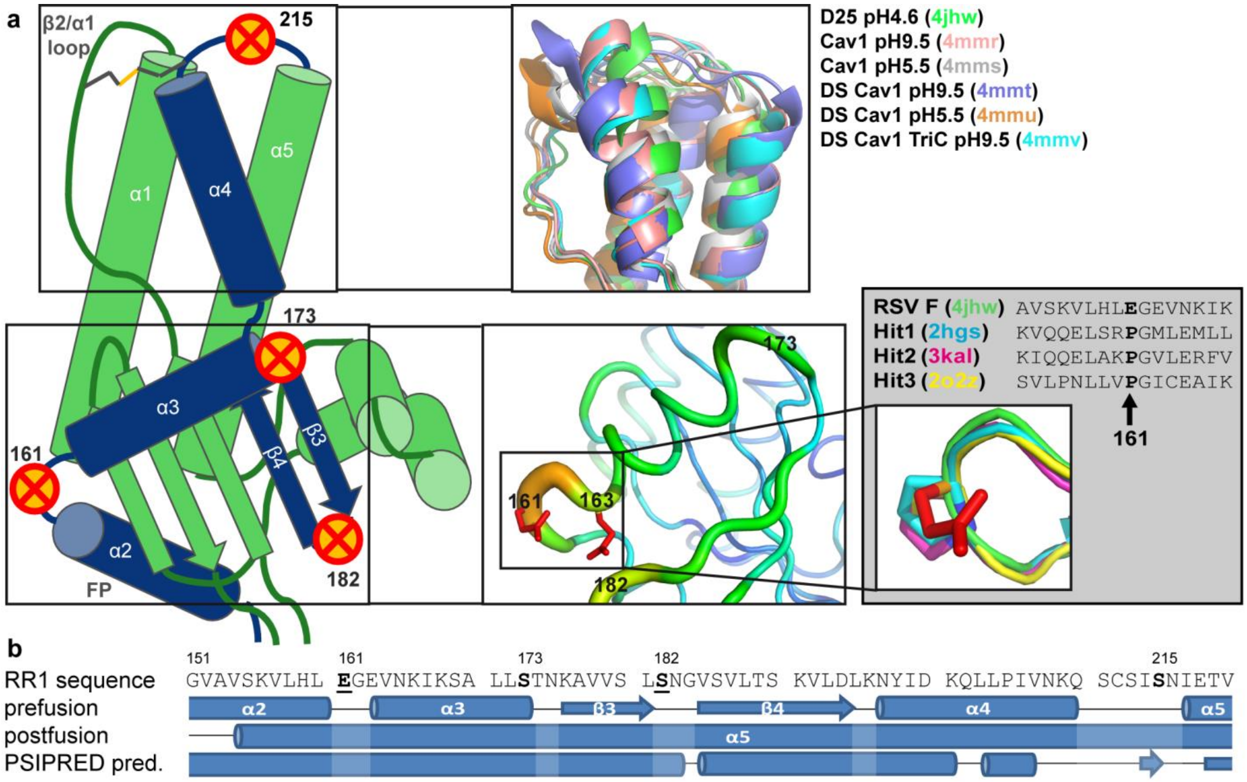

Supplementary Figure 2.

Preventing hinge movements. To guide the exact placement of the prolines in the turns, the Protein Data Bank (PDB) was searched for local structural homology of stable turns that contain proline residues. (**a**, upper panels) Superimposition of all known crystal structures of prefusion F illustrating flexibility in the top. (**a**, lower panels) The turn between helix α2 and α3 and the structural homology with loops that contain proline at position homologous to RSV F Glu161. For the region of α2-α3 turn (residues 161-162), a high structural homology was found with a turn in a helix-turn-helix of several proteins that all had a proline at the homologous 161 position. Glu161 was considered unfavorable because of the high B-factor, bad angles and possible electrostatic repulsion with Glu163. According to the alignment with the structural homologues, the substitution of Glu161 by proline was selected to stabilize this turn and prevent it from refolding. (**b**) RSV F RR1 region (residues 137 – 216). Bold residues are substituted to prolines in Figure 2 and 3. Under the sequence the secondary structure elements as it occurs in the crystal structures of prefusion F and postfusion F are indicated (β-strand as arrows and α-helix as cylinders). The bottom line indicates the region with the highest helix prediction based on PSIPRED (http://bioinf.cs.ucl.ac.uk/psipred/). Underlined residues have bad angles according to Ramachandran-plot.

a

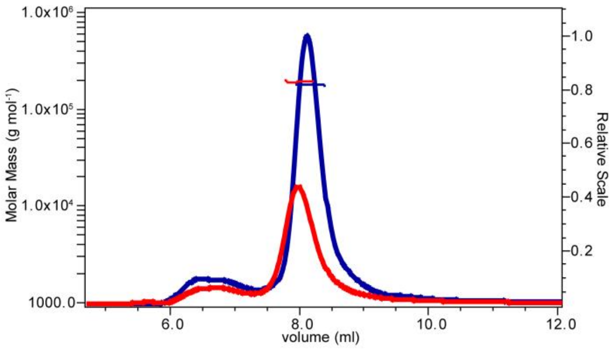

b

Sample	peak 1		peak 2	
	MW (kDa)	Area (%)	MW (kDa)	Area (%)
SC-DM	188	83.1	821	16.2
SC-TM	175	85.5	641	14.0

c

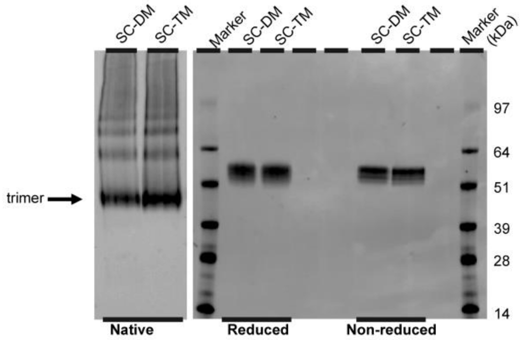

d

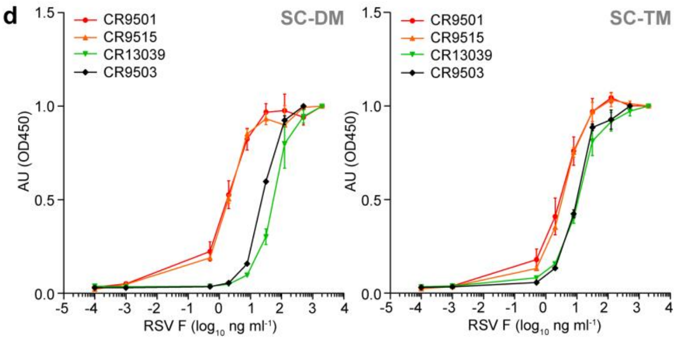

e

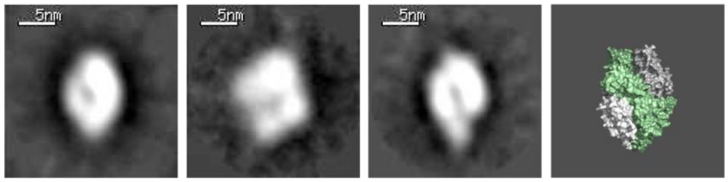

f

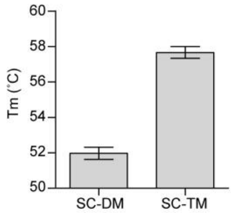

Supplementary Figure 3.

Biochemical analysis of pre-fusion F protein. (**a**) SEC-MALS analysis of SC-DM (red) and SC-TM (blue) with UV trace and (**b**) measured Mw. (**c**) Purified SC-DM and SC-TM were analyzed using NativePAGE (left) and SDS-PAGE (right, reduced and non-reduced) followed by Western blot with RSV-F specific antibody. NativePAGE shows a trimeric RSV F and the reduced SDS-PAGE shows single chain proteins (SC-DM and SC-TM). (**d**) Pre-fusion conformation of the trimeric F proteins SC-DM and SC-TM was confirmed by binding to pre-fusion-specific antibodies CR9501, CR9515 and CR13039 in an ELISA assay(n=3, mean±SEM, two technical replicates in each measurement). MAb CR9503 was used as a conformation independent positive control (n=1, two technical replicates). (**e**) 2D class averaging of electron microscopy images collected with purified RSV F SC-TM. The first three panels show the three major classes of the 2D class averaging. The fourth panel shows a surface representation of the pre-fusion RSV F SC-TM with one protomer shown in green and the other two protomers of the trimer in light and dark grey, respectively. (**f**) thermo-stability of SC-DM and SC-TM using DSF(data presented as mean ± range of 2-3 technical replicates).

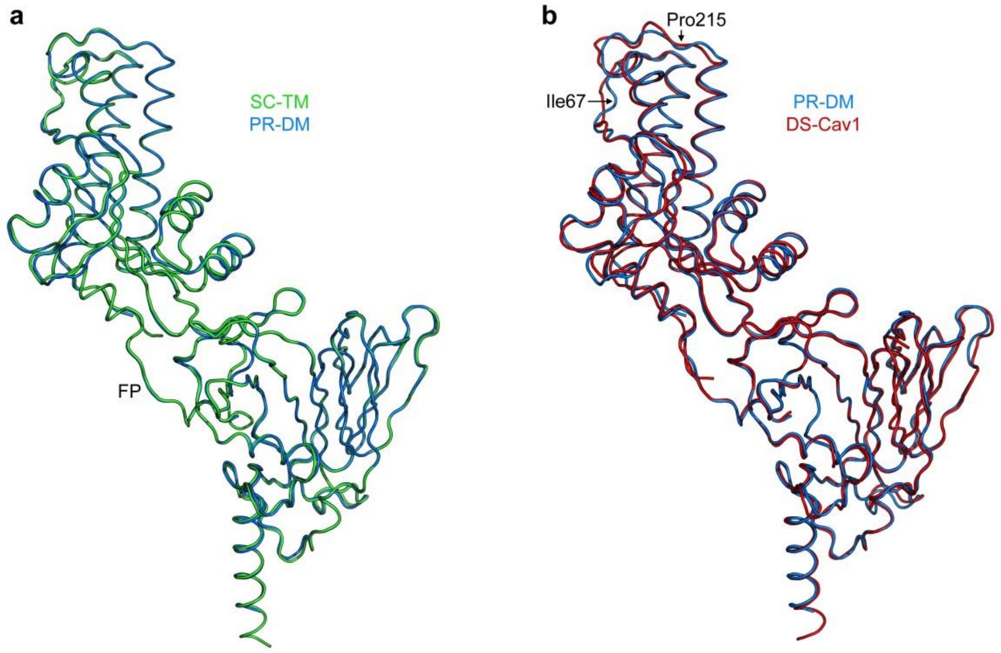

Supplementary Figure 4.

Structural comparison of prefusion-stabilized proteins. (**a**) Superpositions of SC-TM with PR-DM and (**b**) PR-DM with DS-Cav1. Ribbon representations are shown, with SC-TM colored green, PR-DM colored blue, and DS-Cav1 (PDB ID: 4MMU) colored red. The fusion peptide (FP) is labeled, as well as the stabilizing mutations Ile67 and Pro215. An example of the electron density map can be found in **Supplementary Fig. 6**.

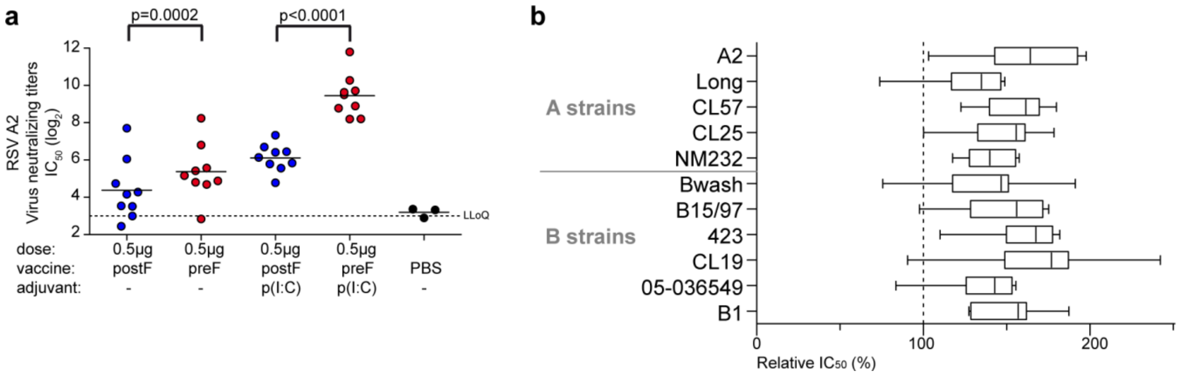

Supplementary Figure 5.

Prefusion RSV F protein is immunogenic in Balb/c mice and immunization of Cotton Rats elicits cross-neutralizing antibodies against various RSV strains. (**a**) Female Balb/c mice (n=9/group) were immunized intramuscularly (i.m.) with 0.5 µg stabilized prefusion RSV F protein (SC-DM) or RSV F protein in postfusion conformation with or without polyI:C at weeks 0, 4 and 8. Mice receiving PBS were used as a control (n=3). Serum was collected at week 12 and VNA performed using RSV A2 as described in Materials and Methods. Horizontal lines indicate the means. Groups were statistically compared as described in the Materials and Methods section. Comparison across doses is indicated by horizontal lines above the groups. Significance was accepted at the p<0.05 level and p-values below 0.05 are indicated in the figure. (**b**) Sera from cotton rats (*Sigmodon hispidus*) (n=8 per group) immunized with prefusion RSV F protein adjuvanted with AdjuPhos were used in a virus neutralization assay (VNA) with the indicated RSV strains. Synagis (palivizumab) at a concentration of 1.072 µg ml^{-1} was used in the assay as a control and the average neutralization titer was set at 100% (vertical dotted line). All values were normalized against this activity for comparison and each group is represented in box and whisker plots, in which the box marks the interquartile range (containing the middle 50% of measured values), the horizontal line within the box marks the median, and the “whiskers” show the complete range of measured values.

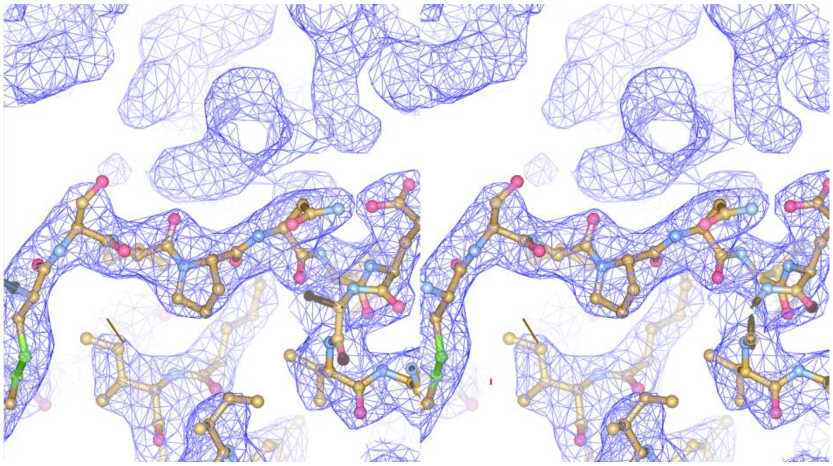

Supplementary Figure 6.

Electron density 2Fo-Fc map contoured at 1 sigma of the α4α5-loop containing S215P.

Strain	Protein ID	Trimerization motif	F1, F2 linker	Termination point	Expression*, (µg/ml)	Fraction of protein binding to CR9501**			Quaternary Structure $
						day 1	day 5	day 30	
B1	$PRsol_{B1}$	None	None	513	5.6	0.4	0.4	0.4	Monomeric
	$PRzl_{B1}$	Isoleucine zipper (L)	None	501	0.7	0.0	ND	ND	NA
	$PRzs_{B1-S}$	Isoleucine zipper (S)	None	495	1.7	0.3	0.1	0.2	monomeric
	$PRzs_{B1}$	Isoleucine zipper (S)	None	513	1.3	0.0	ND	ND	NA
	$PRgcn_{B1}$	GCN4	none	516	2.9	0.0	ND	ND	NA
	PR_{B1-S}	Fibritin	None	499	1.3	0.0	ND	ND	NA
	PR_{B1}	Fibritin	None	513	2.0	0.3	0.0	ND	trimeric
	$PRfko_{B1}$	Fibritin	furin site KO	513	1.0	0.0	ND	ND	NA
	SC_{B1}	Fibritin	GS linker	513	11.6	0.4	0.1	0.0	trimeric
	$SCzs_{B1}$	Isoleucine zipper (S)	GS linker	513	1.1	0.0	ND	ND	NA
	$SCsol_{B1}$	None	GS linker	513	14.0	0.5	0.4	0.4	monomeric
A2	$PRsol_{A2}$	None	None	513	3.6	0.5	0.6	0.5	monomeric
	$SCzs_{A2}$	Isoleucine zipper (S)	GS linker	495	2.0	0.7	0.7	0.6	monomeric
	SC_{A2}	Fibritin	GS linker	513	4.5	0.2	0.1	0.0	mixed monomer/trimer

Supplementary Table 1.

Expression level and prefusion conformation stability of single chain and processed F with and without (sol) trimerization domain. Details on the sequences are explained in **Supplementary Note 1**. *Protein expression levels measured in supernatants of transiently transfected HEK293 cells 96 hours post transfection based on binding to antibody CR9503 as described in Material and Methods, (n=2). **Fraction of RSV F protein binding to pre-fusion specific CR9501 antibody[21] as described in Material and Methods, (n=2). $ Quaternary structure based on Native Page (**Supplementary Fig. 1**)

Supplementary Note 1 Sequences alignment of the different designs for strain B1.

Linker designs:

	110	120	130	140		
WT$_{B1}$	PAANN**R**A**RR**E	LPRFMNYTLN	NAKKTNVTLS	KK**R**K**RR**FLGF	LLGVG	**Furin cleavage recognition motif**
PRfko$_{B1}$	PAANN**Q**ARRE	APQYMNYTIN	TTKNLNVSIS	KKRK**SS**FLGF	LLGVG	**Furin cleavage KO**
SC$_{B1}$	PAANNQAR			**GSGSGR**SLGF	LLGVG	**GS linker**

C-terminal designs:

	500	510	520	530	540	550	560	570	
WT$_{B1}$	SISQVNEKIN	QSLAFIRRSD	ELLHNVNTGK	STTNIMITT*I*	*IIVIIVVLLS*	*LIAIGLLLYC*	KAKNTPVTLS	KDQLSGINNI	AFSK
PRsol$_{B1}$	SISQVNEKIN	QSLAFIRRSD	ELL						
PRsl$_{B1}$	SISQVNEKIN	Q**IEKKIEAIE**	**KKIEAIEKKI**	**EAIEKKIEA**		**Isoleucine Zipper L**			
PRzs$_{B1-S}$	SISQ------	--**EKKIEAIE**	**KKIEAIEKKI**	**EA**		**Isoleucine Zipper S**			
PRzs$_{B1}$	SISQVNEKIN	QSLAFIRRSD	ELLHN**IEKKI**	**EAIEKKIEAI**	**EKKIEA**	**Isoleucine Zipper S**			
PRgcn$_{B1}$	SISQVNEKIN	QSLAFIRRSD	ELLHNV**EDKI**	**EEILSKIYHI**	**ENEIARIKKL**	**IGEA** **GCN4**			
PR$_{B1-S}$	SISQVNEKIS	SLQGDVQALQ	EA-----**GYI**	**PEAPRDGQAY**	**VRKDGEWVLL**	**STFL** **Fibritin**			
PR$_{B1}$	SISQVNEKIN	QSLAFIRRSD	ELLSAIG**GYI**	**PEAPRDGQAY**	**VRKDGEWVLL**	**STFL** **Fibritin**			
